# Additional Personal

**Published:** 1898-11

**Authors:** 


### ADDITIONAL PERSONAL.

CHARLES BENJAMIN KNOWLTON, M. D., of the Buffalo bar and an alumnus of the Buffalo University Medical College, has recently established law offices in the Erie County Savings Bank Building.

